# Patients with Neurodegenerative Proteinopathies Exhibit Altered Tryptophan Metabolism in the Serum and Cerebrospinal Fluid

**DOI:** 10.1021/acschemneuro.3c00611

**Published:** 2024-01-09

**Authors:** Michal Kaleta, Eva Hényková, Kateřina Menšíková, David Friedecký, Aleš Kvasnička, Kateřina Klíčová, Dorota Koníčková, Miroslav Strnad, Petr Kaňovský, Ondřej Novák

**Affiliations:** † Laboratory of Growth Regulators, Institute of Experimental Botany of the Czech Academy of Sciences & Palacky University, Šlechtitelů 27, 783 71 Olomouc, Czech Republic; ‡ Department of Neurology, University Hospital Olomouc, 779 00 Olomouc, Czech Republic; § Department of Neurology, Faculty of Medicine and Dentistry, Palacky University, 779 00 Olomouc, Czech Republic; ∥ Laboratory for Inherited Metabolic Disorders, Department of Clinical Biochemistry, University Hospital Olomouc and Faculty of Medicine and Dentistry, Palacky University Olomouc, Zdravotníků 248/7, 779 00 Olomouc, Czech Republic

**Keywords:** Tryptophan metabolic pathway, neurodegenerative disease, Parkinson’s disease, Alzheimer’s disease, serum, cerebrospinal fluid

## Abstract

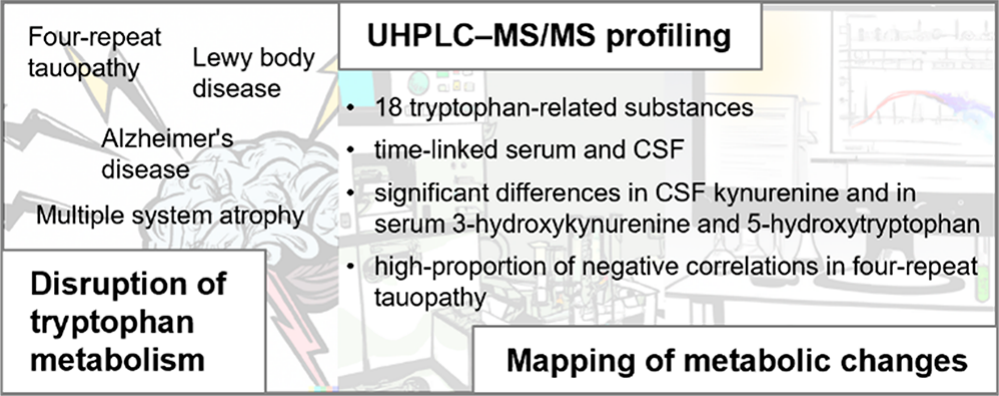

Some pathological conditions affecting the human body can also disrupt metabolic pathways and thus alter the overall metabolic profile. Knowledge of metabolic disturbances in specific diseases could thus enable the differential diagnosis of otherwise similar conditions. This work therefore aimed to comprehensively characterize changes in tryptophan metabolism in selected neurodegenerative diseases. Levels of 18 tryptophan-related neuroactive substances were determined by high throughput and sensitive ultrahigh-performance liquid chromatography–tandem mass spectrometry in time-linked blood serum and cerebrospinal fluid samples from 100 age-matched participants belonging to five cohorts: healthy volunteers (*n* = 21) and patients with Lewy body disease (Parkinson’s disease and dementia with Lewy bodies; *n* = 31), four-repeat tauopathy (progressive supranuclear palsy and corticobasal syndrome; *n* = 10), multiple system atrophy (*n* = 13), and Alzheimer’s disease (*n* = 25). Although these conditions have different pathologies and clinical symptoms, the discovery of new biomarkers is still important. The most statistically significant differences (with *p*-values of ≤0.05 to ≤0.0001) between the study cohorts were observed for three tryptophan metabolites: l-kynurenine in cerebrospinal fluid and 3-hydroxy-l-kynurenine and 5-hydroxy-l-tryptophan in blood serum. This led to the discovery of distinctive correlation patterns between the profiled cerebrospinal fluid and serum metabolites that could provide a basis for the differential diagnosis of neurodegenerative tauopathies and synucleinopathies. However, further large-scale studies are needed to determine the direct involvement of these metabolites in the studied neuropathologies, their response to medication, and their potential therapeutic relevance.

## 1 Introduction

The kynurenine pathway plays a key role in l-tryptophan (TRP) metabolism and is the source of many substances essential for the human body.^1,2^ In mammals, the majority (∼95%) of ingested TRP is metabolized via this route.^3,4^ The products of this pathway, the so-called kynurenines, include both neurotoxic substances, such as 3-hydroxykynurenine (3-OH-KYN) and neuroprotective substances, such as kynurenic acid (KA). The remaining minor fraction of ingested TRP is metabolized via the methoxyindole, kynuramine, and intestinal bacterial indole pathways.^2,5−7^ Kynurenine metabolites have been linked to a number of important physiological processes including inflammation, immune responses, and neurotransmission.^3^ It has also been suggested that disruption of the kynurenine metabolic pathway contributes significantly to the development of metabolic syndrome, Parkinson’s disease (PD), and Alzheimer’s disease (AD).^8^ Moreover, there is evidence that the initial enzymes of this pathway (hepatic tryptophan 2,3-dioxygenase, EC 1.13.11.11; extrahepatic indoleamine 2,3-dioxygenase, EC 1.13.11.52) are stimulated by glucocorticoids and proinflammatory cytokines, prompting suggestions that it is activated preferentially during chronic stress and infection.^9^ Under normal conditions, these enzymes are expressed weakly and only in certain areas of the brain.^10^ The activity of the kynurenine pathway in the brain therefore depends mainly on the transport of l-kynurenine (KYN) and 3-OH-KYN from peripheral sources across the blood–brain barrier. However, not all kynurenine pathway metabolites are equally able to cross the blood–brain barrier, so dysregulation of kynurenine metabolism in the periphery and the central compartment can have different functional consequences.^8^

Unsurprisingly, most research in this area has focused on the two most common neurodegenerative diseases: PD and AD.^11^ Changes in kynurenine metabolism have been characterized in some detail in both PD^8,10−20^ and AD,^1,8,10,21−23^ revealing some notable characteristic trends. First, PD patients exhibit reduced plasma^16,20^ and serum^8,10,12,17,19^ concentrations of TRP relative to controls. Reduced serum TRP levels may be associated with the psychiatric problems that occur in PD patients.^19^ Increased degradation of TRP in peripheral blood leading to reduced serum TRP levels has also been observed in AD.^21^ Additionally, some observations indicate that PD patients have reduced KYN levels in both plasma^16^ and serum^11,17^ together with elevated KYN levels in the cerebrospinal fluid (CSF).^15^ Moreover, several authors have reported elevated levels of 3-OH-KYN in diverse biological matrices of PD patients, including serum,^11,17^ plasma,^14^ and CSF.^13,15^ However, Oxenkrug et al. (2017) reported that serum KYN concentrations in PD patients were higher than in a control group.^8^ These authors were unable to determine the levels of 3-OH-KYN because of the low sensitivity of their chosen analytical method. Other metabolic changes observed are described in the Discussion section.

Conventional methods for diagnosing neurodegenerative diseases are mainly based on brain imaging but have been enhanced in recent years by the possibility of monitoring various predictive, prognostic, or diagnostic biomarkers, especially high molecular weight protein biomarkers.^24,25^ There are several established biomarkers for neuropathologies, and new ones have been proposed.^24,26,27^ Changes in the levels of low molecular weight neurotransmitter metabolites in the serum,^8,10−12,17,19,21,22^ plasma,^1,14,16,18,20^ and CSF^1,10,12−15,18,20,23^ of AD and PD patients have also been studied in detail. However, metabolic dysregulation of low molecular weight metabolites is comparatively understudied, particularly in less common neuropathologies, and therefore warrants further investigation.

In this study, we used a highly efficient and high-throughput ultrahigh-performance liquid chromatography–tandem mass spectrometry (UHPLC–MS/MS) method for metabolic profiling of 18 TRP-related substances,^2^ including metabolites of the kynurenine, methoxyindole, and tryptamine and indoles pathways (see Figure S1). We analyzed these substances in time-matched serum and CSF from the same participant in healthy control (HC) group and patient cohorts with PD, dementia with Lewy bodies (DLB), progressive supranuclear palsy (PSP), corticobasal syndrome (CBS), multiple system atrophy (MSA), and AD. The PD and DLB clinical units were combined into the Lewy body disease (LBD) group and the PSP and CBS units into the four-repeat tauopathy (4R-Tau) group. Our basic hypothesis was that the levels of these metabolites may be altered by certain pathological processes that affect the nervous system. The aim of our study was therefore to comprehensively quantitate a wide set of TRP metabolites spanning several metabolic pathways in parallel in two compartmentally separated biological fluids. We also analyzed biological samples representing several pathological conditions of the nervous system. This is notable because most previously reported studies have had a much narrower focus, examining only a few analytes and often only a single sample type. Additionally, the available literature data on different metabolites and biological matrices are derived from a wide range of analytical methods, which can result in inconsistent outputs that make it difficult to draw meaningful conclusions in comparing different conditions. We therefore aimed to comprehensively map changes in every TRP metabolic pathway in multiple neuropathologies using a single highly selective and robust analytical method whose high sensitivity enables the mapping of analytes at femtomolar levels.

## 2 Results

Eighteen TRP metabolites were analyzed in blood serum and CSF samples representing selected neurodegenerative proteinopathies using a UHPLC–MS/MS-based method. The concentrations of eight analytes (*N*-methylserotonin, *N*-Me-S; tryptamine, TA; *N*-methyltryptamine, *N*-Me-TA; 5-methoxytryptamine, 5-MeO-TA; *N*-acetylserotonin, *N*-Ac-S; 6-hydroxymelatonin, 6-OH-M; melatonin, M; *N*^1^-acetyl-*N*^2^-formyl-5-methoxykynuramine, AFMK) were below the limit of detection or quantification in all or most participants and were therefore excluded from the statistical evaluation. The remaining 10 analytes (TRP; 3-OH-KYN; serotonin, S; KYN; 5-hydroxy-l-tryptophan, 5-OH-TRP; 3-hydroxyanthranilic acid, 3-OH-AA; 5-hydroxyindole-3-acetic acid, 5-OH-IAA; KA; anthranilic acid, AA; indole-3-acetic acid, IAA) could be quantified and were included. For those samples in which the concentrations of selected analytes were below the limit of quantification, missing values were imputed using the *k*-nearest neighbors algorithm.^28,29^ The original data were tested for normality, which was achieved after log transformation.

**Figure 1:**
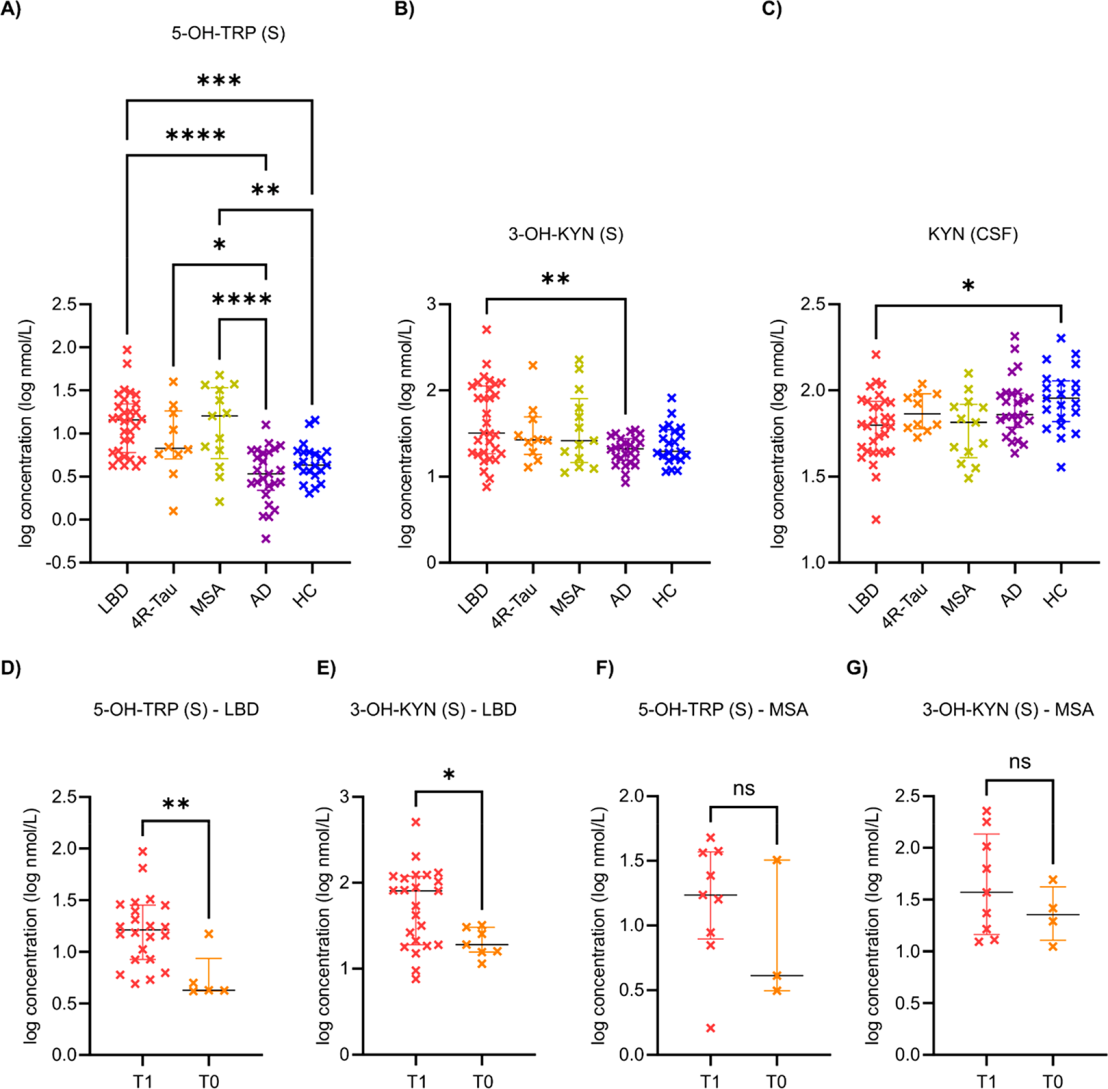
Serum 5-hydroxy-l-tryptophan (5-OH-TRP; A), serum 3-hydroxy-l-kynurenine (3-OH-KYN; B), and CSF kynurenine (KYN; C) concentrations in the Lewy body disease (LBD; *n* = 31) four-repeat tauopathy (4R-Tau; *n* = 10), multiple system atrophy (MSA; *n* = 13), Alzheimer’s disease (AD; *n* = 25), and healthy control (HC; *n* = 21) groups. Serum levels of 5-OH-TRP (D, F) and 3-OH-KYN (E, G) in treated (T1) and untreated (T0) LBD (D, E) and MSA (F, G) patients. Data are expressed as log of transformed concentrations (log nmol/L). Asterisks ∗, ∗∗, ∗∗∗, and ∗∗∗∗ denote *p*-values of ≤0.05, ≤0.01, ≤0.001, and ≤0.0001, respectively.

Parametric ANOVA with post hoc testing by the Holm–Shidak multiple comparison test was used to compare the study groups. The serum 5-OH-TRP (Figure 1A) concentration in the HC group differed significantly from those in the LBD (*p* = 0.000 12) and MSA groups (*p* = 0.007 22), while that of the AD group differed significantly from those in the LBD (*p* < 0.000 01), 4R-Tau (p = 0.021 49), and MSA (*p* = 6 × 10^–5^) groups. The serum 3-OH-KYN (Figure 1B) concentrations of the LBD and AD groups also differed significantly (*p* = 0.004 07), as did the CSF KYN (Figure 1C) concentrations of the LBD and HC groups (*p* = 0.019 17). Here it should be noted that the statistical significance of the observed differences depends heavily on the number of samples in each group being compared. The effects of treatments on TRP metabolite concentrations were also evaluated using the Mann–Whitney *U* test in the LBD (Figure 1D,E) and MSA (Figure 1F,G) patient groups. Treatment significantly increased the concentrations of 5-OH-TRP (*p* = 0.0037) and 3-OH-KYN (*p* = 0.0373) in LBD patients compared to dopaminergic untreated patients. Similar trends existed in the MSA group, but statistical significance was not achieved in this case due to the limited number of samples.

**Figure 2:**
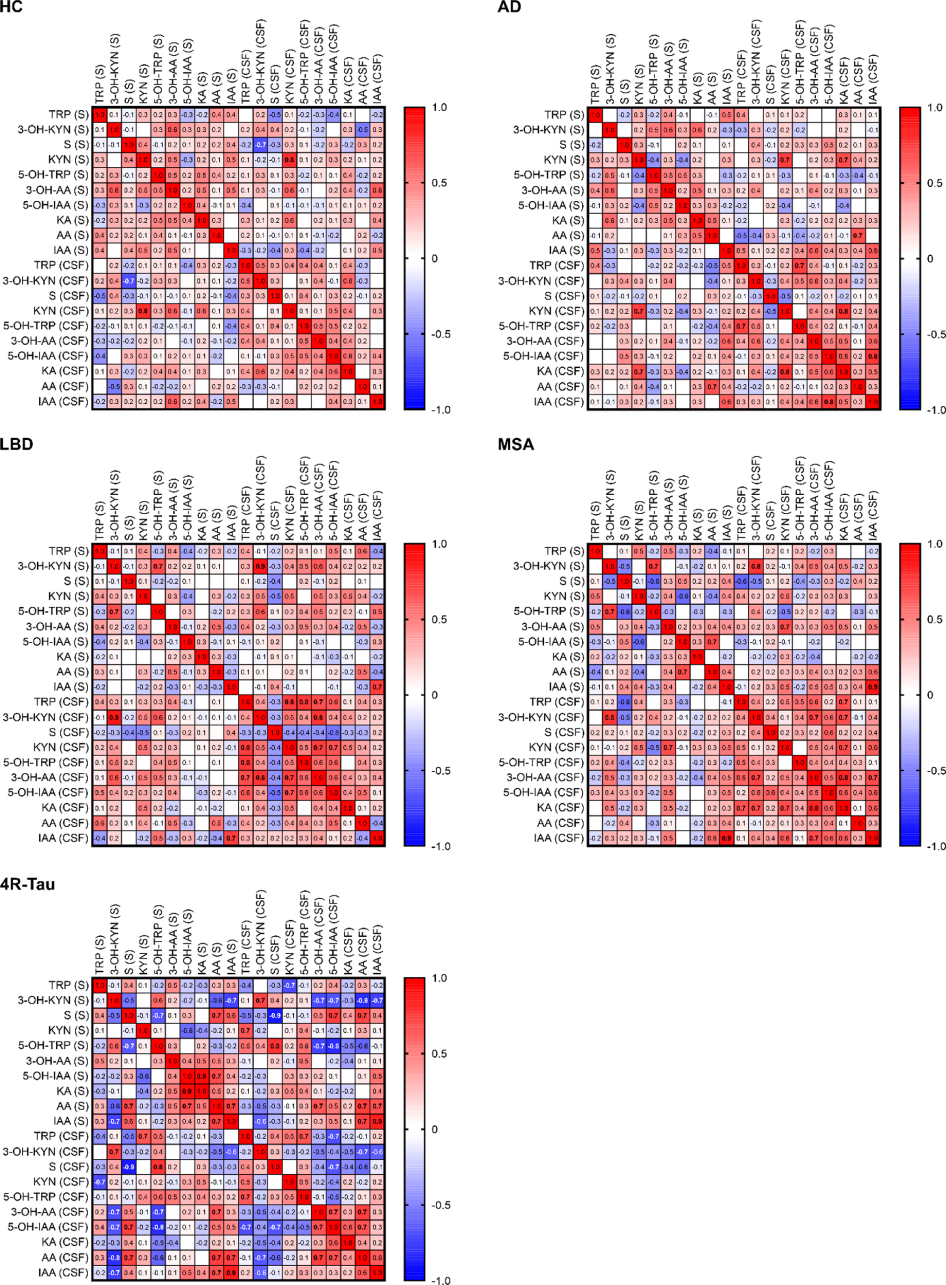
Pearson correlation heatmaps of selected tryptophan metabolites in the healthy control (HC; *n* = 21), Alzheimer’s disease (AD; *n* = 25), Lewy body disease (LBD; *n* = 31), multiple system atrophy (MSA; *n* = 13), and four-repeat tauopathy (4R-Tau; *n* = 10) groups. Strong significant positive and negative correlations (*r* ≥ 0.7 or *r* ≤ −0.7) are marked in bold. Red and blue fields correspond to positive and negative correlations, respectively. The metabolites are denoted in accordance with the list of abbreviations.

The relationships between the concentrations of 10 analytes in the serum and the CSF of each patient group were evaluated based on Pearson correlations. Heat maps of these correlations are presented in Figure 2, where red and blue fields correspond to positive and negative correlations, respectively, and the strengths of the correlations are indicated by the intensity of the coloration and shown explicitly by using numbers. Several statistically significant strong, moderate, and weak correlations were found, and there were some clearly different trends within the studied groups. The HC group exhibited only one strong correlation: serum KYN concentrations correlated positively with those in the CSF (*r* = 0.80). The AD, LBD, and MSA groups had a wider range of positive correlations. In AD patients, there were strong positive correlations between the concentrations of KYN in the serum and CSF (*r* = 0.74), TRP and 5-OH-TRP in CSF (*r* = 0.74), KYN and KA in CSF (*r* = 0.79), and 5-OH-IAA and IAA in CSF (*r* = 0.77). The LBD group exhibited strong positive correlations between the serum and CSF concentrations of 3-OH-KYN (*r* = 0.89) and IAA (*r* = 0.75). In addition, there were strong positive correlations between the concentrations of several metabolites within the CSF, including TRP and KYN (*r* = 0.75), TRP and 5-OH-TRP (*r* = 0.76), 3-OH-KYN and 3-OH-AA (*r* = 0.78), and KYN and 3-OH-AA (*r* = 0.75). MSA samples exhibited strong positive correlations between serum 3-OH-KYN and 5-OH-TRP (*r* = 0.74), serum and CSF 3-OH-KYN (*r* = 0.78), serum and CSF IAA (*r* = 0.89), CSF 3-OH-KYN and 3-OH-AA (*r* = 0.72), and CSF 3-OH-AA and KA (*r* = 0.83). Interestingly, the 4R-Tau group differed significantly from the others in that it had many negative correlations (Figure 2), including strong negative correlations between serum TRP and CSF KYN (*r* = −0.70), serum 3-OH-KYN and IAA (*r* = −0.73), serum 3-OH-KYN and CSF 3-OH-AA (*r* = −0.70), serum 3-OH-KYN and CSF AA (*r* = −0.81), serum 3-OH-KYN and CSF IAA (*r* = −0.74), serum and CSF S (*r* = −0.88), serum 5-OH-TRP and CSF 3-OH-AA (*r* = −0.73), and serum 5-OH-TRP and 5-OH-IAA (*r* = −0.82).

## 3 Discussion

This study comprehensively mapped changes in TRP metabolism via the kynurenine, methoxyindole, kynuramine, and intestinal bacterial indole pathways in time-matched CSF and serum samples from patient groups representing four degenerative neuropathologies: AD, LBD, MSA, and 4R-Tau. Such comprehensive mappings are valuable because monitoring of specific metabolic changes (i.e., changes in the levels of selected biomarkers) could facilitate the differential diagnosis of these disease states. We found no statistically significant between-cohort differences in the concentrations of TRP S, 3-OH-AA, 5-OH-IAA, KA, AA, and IAA in either the serum or the CSF. However, significant differences were observed for the serum concentrations of 5-OH-TRP and 3-OH-KYN as well as the CSF concentration of KYN.

Preliminary data indicate that 5-OH-TRP improves global sleep quality in patients with PD and REM sleep behavior disorder, which are often associated with each other.^30^ Moreover, 5-OH-TRP supplementation reportedly reduced depressive symptoms in PD^31^ and significantly reduced L-DOPA-induced dyskinesia in PD.^32^ Earlier studies examined the use of this aromatic amino acid in the treatment of depression^33,34^ and showed that levels of 5-OH-TRP in Alzheimer-type dementia CSF samples were lower than in matched controls.^23^ Additionally, Havelund et al. (2017) reported that 5**-**OH-TRP levels in blood plasma from PD patients receiving L-DOPA (dyskinetic, *n* = 10; nondyskinetic, *n* = 8) were roughly twice those in PD patients not receiving L-DOPA (*n* = 8) and controls (*n* = 14).^18^ The authors attributed this to the fact that PD patients are treated with peripheral decarboxylase inhibitors and L-DOPA, which is a substrate of aromatic amino acid decarboxylase (EC 4.1.1.28; DOPA decarboxylase; AADC), the enzyme that catalyzes the metabolic conversion of 5-OH-TRP into S. Substrate competition between L-DOPA and 5-OH-TRP at AADC could thus reduce the rate of 5-OH-TRP conversion and increase its concentration in the body. We found that serum 5-OH-TRP levels were significantly higher in the LBD group (i.e., patients with PD and DLB) than in the HC and AD groups (Figure 1A). Most LBD patients (24 out of 31) were also taking some form of L-DOPA and peripheral decarboxylase inhibitors at the time of blood and CSF sampling. Dividing the LBD group into medicated and unmedicated patients revealed that serum 5-OH-TRP concentrations were significantly higher in patients receiving antiparkinsonian drugs than in those not receiving such treatment (Figure 1D). Our findings thus agree with those of Havelund et al. (2017).

A similar increase in 5-OH-TRP levels was observed in the 4R-Tau and MSA groups (Figure 1A). The limited number of 4R-Tau patients meant that this group could not be further divided to evaluate the effect of treatment, and no statistically significant treatment effect was observed for the MSA group, although it should be noted that this group had a very small number of untreated patients (Figure 1F). Despite the apparently similar trends in these groups, the origin of the elevated 5-OH-TRP levels in MSA may differ from that in LBD patients. There have been comparatively few studies on metabolic changes in 4R-Tau or MSA, but some works have measured concentrations of polyamines (e.g., putrescine, cadaverine, spermidine),^35^ catechols (dopamine, norepinephrine, L-DOPA, dihydroxyphenylacetic acid, and dihydroxyphenylglycol),^36^ selected amino acids (l-glutamate, l-arginine, and l-citrulline levels),^37^ polyunsaturated fatty acids (e.g., arachidonic acid, eicosapentaenoic acid, docosahexaenoic acid),^38^ nitrate,^39^ coenzyme Q10,^40^ and glutathione^41^ in MSA patients. Additionally, Kaiserova et al. (2021) analyzed 5-OH-IAA in the CSF of patients with PD, MSA, PSP, and CBS,^42^ revealing that levels of this metabolite did not differ significantly from controls in the tauopathies PSP and CBS but were significantly reduced in the synucleinopathies PD and MSA. The authors suggested that this may be because synucleinopathies cause more severe damage to the serotogenic system. While some larger metabolic studies^43,44^ with broader scopes have been reported, we are not aware of any earlier studies that have comprehensively mapped TRP metabolism or any of its individual pathways in MSA and 4R-Tau.

Drugs are not the only factors that may affect TRP metabolite levels in the studied pathologies. For example, the elevated 5-OH-TRP concentrations in PD patients may result from other metabolic changes such as reduced metabolic conversion of 5-OH-TRP. This possibility is supported by the results of Nagatsu and Sawada (2007), who found that the activities and/or mRNA and protein levels of AADC and other enzymes are reduced in the brains of human PD patients.^45^ Similarly, Tehranian et al. (2006) observed inhibition of AAAD enzyme activity in dopaminergic cells overexpressing alpha-synuclein.^46^ The authors attributed this effect to interactions between AADC and α-synuclein, which forms in Lewy bodies and Lewy neurites during PD. Dietary factors may also influence TRP metabolite levels because TRP is an essential amino acid that humans cannot biosynthesize.^47^ The relationship between dietary TRP consumption and its levels in the body will thus affect the levels of its derived metabolites. Further work is needed to determine whether the increased 5-OH-TRP levels observed in various proteinopathies are mainly due to medication (e.g., use of L-DOPA), the pathological process itself, or a combination of these factors.

Our results also suggest that serum 3-OH-KYN could be a target for the treatment of the neurodegenerative diseases examined in this study. This metabolite is known to be neurotoxic and to induce mitochondrial dysfunction and cell death via free radical generation and oxidative stress, possibly in synergy with the excitotoxin quinolinic acid.^14^ Free radical generation and increased oxidative activity both cause neuronal damage,^11^ suggesting that 3-OH-KYN may be involved in the pathogenesis of PD. This suggestion is supported by clinical observations and multiple genome-wide association studies that have revealed an association between neurodegeneration and changes in the kynurenine pathway.^14^ Our results showed that LBD (PD and DLB) patients had significantly higher serum levels of 3-OH-KYN than AD patients (Figure 1B) and exhibited a similar but nonsignificant increase relative to the HC cohort. This finding is consistent with previous studies reporting the dysregulation of the kynurenine pathway in PD patients. For example, Heilman et al. (2020) found that plasma levels of 3-OH-KYN were significantly elevated in PD patients, most of which did not exhibit dyskinesia.^14^ The authors attributed this to reduced activity of the enzyme kynureninase (EC 3.7.1.3), which catalyzes the conversion of 3-OH-KYN into 3-OH-AA. This hypothesis is consistent with the reduced plasma levels of 3-OH-AA observed in their study and, together with the other findings mentioned above, suggests that 3-OH-KYN could serve as a plasma biomarker of PD severity and/or progression. The development of such a biomarker could obviate the need for CSF sampling, which would greatly benefit patients because obtaining blood samples is easier and also less risky and invasive than collecting CSF by a lumbar puncture. Elevated plasma levels of 3-OH-KYN have also been observed in PD patients with dyskinesia who were being treated with L-DOPA,^18^ in accordance with other reports.^11,17^ Similarly increased levels of this strong excitotoxin have also been observed in the CSF of PD patients^13,15^ and in certain brain regions in PD, namely, the *putamen*, *prefrontal cortex*, and *pars compacta* of the *substantia nigra*.^48^ However, we observed no significant changes in the CSF concentration of 3-OH-KYN. This is consistent with an earlier study^14^ in which it was suggested that the differing reported trends in the serum and CSF concentrations of 3-OH-KYN may be due to differences in its production or metabolism in the peripheral and central compartments. The ratio of 3-OH-KYN and KA was also significantly increased in the CSF of PD patients, which supports a proposed therapeutic strategy based on blocking the production of excitotoxic 3-OH-KYN and promoting the synthesis of neuroprotective KA.^13^ Disruption of the kynurenine pathway could contribute to the clinical progression of PD because some of its metabolites increase oxidative stress and cytokine-mediated neuroinflammation in the CNS,^15^ which again suggests that 3-OH-KYN could be a good therapeutic target for PD treatment. However, it is still important to consider whether elevated 3-OH-KYN levels are a cause of PD.

We also considered the possibility that serum 3-OH-KYN levels could be affected by specific therapies because previous studies have demonstrated that certain medications (e.g., antidepressants and L-DOPA) can alter the concentrations of kynurenine pathway metabolites.^10,18^ The published data on this issue are somewhat contradictory, however, because Sorgdrager et al. (2019) found that medication did not affect the levels of six kynurenine pathway metabolites (TRP, KYN, 3-OH-KYN, KA, xanthurenic acid, and quinolinic acid) in PD and AD.^10^ Similarly, Oxenkrug et al. (2017) found no differences between untreated and L-DOPA treated PD patients with respect to the plasma levels of TRP, KYN, AA, KA, and 3-OH-KYN, and therefore did not further stratify these patient groups.^8^ Nevertheless, we found that therapy influenced 3-OH-KYN levels in LBD and MSA patients (Figure 1E,G), although a statistically significant effect was observed only for the LBD group; the failure to reach significance for the MSA group may be due to its low number of samples. The evidence that established anti-PD treatments may increase levels of neurotoxic 3-OH-KYN suggests that complementary treatments targeting this metabolite could be valuable in the management of PD.

Another interesting metabolite is KYN, whose concentration in the CSF of untreated patients with PD (*n* = 16) was significantly lower than in controls (*n* = 16).^49^ A similar reduction was observed previously in PD patients treated with L-DOPA/carbidopa,^50^ although Iwaoka et al. (2020) reported that KYN levels in the CSF of PD patients (*n* = 20; 18 without antiparkinsonian medication, 2 treated with L-DOPA) were significantly higher than in controls (*n* = 13).^15^ We found that the KYN concentrations in the CSF of LBD patients (*n* = 31) were significantly lower than those in the HC group (*n* = 21) (Figure 1C). However, our patient cohorts were larger than those examined by Iwaoka et al. (2020), and we used mass spectrometric rather than electrochemical detection. A similar trend of slightly reduced KYN and KA concentrations was observed in human post-mortem samples of the *prefrontal cortex*, *putamen*, and *substantia nigra* of PD patients,^4,48^ and low KYN concentrations have been found in the plasma of PD patients^16^ and the serum of PD patients.^11,12,17^ However, Oxenkrug et al. (2017) reported elevated serum KYN levels in PD patients.^8^ The inconsistencies between these findings may be partly due to the use of different analytical approaches and differences in the studied cohorts’ clinical characteristics (e.g., patient age, disease duration, severity, and gender representation). It should also be noted that TRP metabolism via the kynurenine pathway may be partly regulated by the gut microbiota, which has important implications for CNS functionality.^51^

Other studies have also looked for differences between several neuropathological cohorts, but most of these studies have focused on the analysis of protein markers. There are studies investigating different patterns of CSF glial markers in DLB and AD patients^52^ or plasma protein biomarkers of neurodegeneration in DLB, AD, frontotemporal dementia, and PSP.^53^ A similar experimental design to our work is described in the work of Lourenco et al. (2021).^54^ They focused on the analysis of a panel of 50 analytes, including neurotransmitters, cytokines, chemokines, and hormones, in the CSF of control participants without dementia and patients with DLB, mild cognitive impairment, and AD. The shared analyte was S, for which, as in our case, no statistically significant changes were observed between patient groups.

Our findings and those reported previously suggest that a testing panel of neuroactive TRP metabolites could have significant diagnostic benefits. For example, the high proportion of negative correlations observed in 4R-Tau samples (see Figure 2) could facilitate the development of a tool for differential diagnosis of neurodegenerative parkinsonism that would distinguish tauopathies (PSP and CBS) from synucleinopathies (DLB, PD, and MSA).^55^ Moreover, it may be possible to link observed differences in the concentrations of the panel metabolites to the differing pathophysiologies of these conditions. Changes in different phases of TRP metabolism have been shown to have differing effects on the potential neurotoxicity of protein aggregates including beta amyloid and pathological alpha-synuclein or tau protein aggregates.^56−59^ Therefore, alterations in specific stages of the kynurenine metabolic pathway could contribute to the development of different neurodegenerative proteinopathies, while also altering the spectrum of TRP metabolites present in CSF or serum. We believe that our discovery of negative correlations between TRP metabolite levels in 4R-Tau patients could be a first step toward the development of tools enabling differential diagnosis of synucleinopathies and tauopathies, but the realization of such a tool will require further elucidation of the influence of biochemical changes in both groups. To this end, it would be very desirable to measure TRP metabolite concentrations in a larger sample set; the resulting data could reveal differences in the concentrations of these metabolites across the spectrum of neurodegenerative proteinopathies and thus provide a robust basis for their differentiation.

It should be emphasized that the result of our work is not the discovery of specific biomarkers but the mapping of trends that occur in the studied neurodegenerative entities. As we suggest, finding effective tools for differential diagnosis will not be possible without a deeper understanding of the close relationship between the pharmacological treatment of given conditions and the metabolic changes that they cause. This issue should definitely be considered in further research. Based on our observations, we believe that a panel of several relevant biomarkers, preferably both low and high molecular weights, will need to be designed to provide a reliable diagnostic tool. In addition, parallel analysis of multiple biofluids may also be very beneficial.

## 4 Conclusion

A major finding of this study is the discovery of condition-specific patterns of correlations between the serum and CSF concentrations of the studied metabolites, which may eventually enable easy discrimination between tauopathies (PSP and CBS) and synucleinopathies (DLB, PD, and MSA). Further testing of larger patient cohorts and longitudinal studies will be needed to identify and validate reliable biomarkers for this purpose. The statistical significance of observed differences depends heavily on the number of samples in each group being compared; therefore, future studies with a larger number of participants will definitely be needed to confirm our results. We are also aware of the fact that the disease progression together with the continuous pharmacological treatment should certainly jeopardize the results of performed CSF examinations; this would deserve future large, double-blind, and long-term studies targeting the candidate metabolite biomarkers.

Notable strengths of this study include the simultaneous metabolic profiling of a relatively high number of analytes (18 metabolites) in time-linked human serum and CSF samples from all participants and the inclusion of multiple nervous system pathologies: LBD, 4R-Tau, MSA, and AD. Previous studies in this area have typically focused on fluctuations in a smaller range of TRP metabolites and only examined PD or AD; to our knowledge, this is the first comprehensive study on the metabolic dysregulation of TRP metabolism in proteinopathies. As this study was only observational and thus provides no basis for inference of direct causal relationships, further research is needed to clarify the relationships described herein. Metabolite levels were not corrected for body mass index, which is a limitation of the study and could be investigated in more detail on larger cohorts of participants in the future. Overall, however, the results obtained show that TRP metabolism is impaired in different ways by various pathological conditions affecting the nervous system and by the pharmacological interventions used to treat these conditions, leading to distinct effects on the concentrations of TRP metabolites in the blood and CSF.

## 5 Material and Methods

### 5.1 Chemicals and Reagents

The deuterated internal standards D_4_-S, D_3_-5-OH-TRP, D_4_-TA, D_5_-TRP, D_4_-5-MeO-TA, D_5_-5-OH-IAA, D_5_-KA, D_4_-AA, D_4_-M, and D_4_-6-OH-M were purchased from C/D/N Isotopes (Canada). D_5_-IAA was obtained from Olchemim Ltd. (Czech Republic), D_6_-KYN was obtained from Cambridge Isotope Laboratories (USA), and [^13^C_2_^15^N_1_]-3-OH-KYN and D_3_-3-OH-AA were obtained from Toronto Research Chemicals (Canada). The internal standards D_3_-*N*-Me-S, D_3_-*N*-Me-TA, D_3_-*N*-Ac-S, and D_3_-AFMK were synthesized using published procedures.^60,61^ Corresponding unlabeled standards, bovine serum albumin, and formic acid were purchased from Sigma-Aldrich (USA). A CSF calibrator was purchased from Tocris Bioscience (UK). All solvents were gradient grade for LC or hypergrade for LC–MS (Merck Millipore, Germany). Argon was obtained from Linde Industrial Gases (Czech Republic). All other used chemicals were purchased from Lachner (Czech Republic).

### 5.2 Study Participants

The study was approved by the ethics committee of the Faculty of Medicine and Dentistry, Palacky University Olomouc, and University Hospital Olomouc. Ethics approval for this study was granted according to University Hospital Olomouc Standard SM-L031 and Ethics Committee Reference Numbers 139/10 and 76/15. All participants were informed of the study’s purpose and design and signed informed consent forms. Blood serum and CSF samples were collected, pretreated, transported, and stored under standardized conditions. Patient recruitment, sample collection, and laboratory analyses were performed between 2016 and 2022. The study was not preregistered.

The study included a total of 100 age-matched male and female participants that were divided into HC and six groups representing the following core clinical entities, each with a different clinical diagnosis and presumed type of neurodegenerative proteinopathy: PD, DLB, PSP, CBS, MSA, and AD. All clinical diagnoses were based on established clinical diagnostic criteria.^62−69^ The patients underwent thorough neurological examination at the tertiary movement disorders center to establish clinical diagnosis; the other (than neurodegenerative) causes of symptoms were carefully excluded. The 1.5 T or 3.0 T magnetic resonance imaging (MRI) of the brain and the dopamine transporter DaTScan (^123^I-ioflupane) imaging were done in all participants, in indicated cases was the positron emission tomography (PET; ^18^F-flutemetamol) brain imaging done as well. All patients were followed up in the tertiary movement disorders center; the final clinical diagnosis was confirmed at the same time when the blood serum and CSF examinations were done.

In all patients, the vascular origin of neurological symptoms, including cognitive deterioration, was excluded using imaging studies: T2-weighted, fluid-attenuated inversion recovery (FLAIR) and diffusion-weighted MRI (DWI-MRI), ultrasonography (USG) and transcranial Doppler (TCD) examinations, and using the calculation of Hachinski ischemic score (HIS); its value in all patients was less than 3.

The PD and DLB clinical units were combined into a single LBD group because of the high similarity of their basic morphological changes in the histopathological findings defined in the pathological diagnostic criteria.^70,71^ For the same reason, the PSP and CBS clinical units were combined in the 4R-Tau group. None of the patients suffering from corticobasal degeneration had a previous diagnosis of frontotemporal dementia. Patients with other serious comorbidities (e.g., hematological disease, cancer, depression, psychosis, chronic kidney disease, or metabolic derangements) were excluded from the study. Behavioral variant frontotemporal dementia (*bv*FTD) is pathologically extremely heterogeneous entity. Current neuropathological classification of degenerative proteinopathies is based on the presence of a predominant pathology. Thus, most cases of FTD are accordingly classified within one of three broad molecular subgroups: frontotemporal lobar degeneration with tau, TDP-43, or FET protein accumulation. Based on the clinical presentation, the relevant pathology cannot be presumed in most cases of *bv*FTD.^72^ This was the principal reason why this disease was excluded from the cohort.

None of the patients nor controls have been treated with corticosteroids; in the HC group, corticosteroid treatment has been one of the exclusion criteria. The HC group consisted of participants examined for benign conditions (e.g., back pain, carpal tunnel syndrome, or tension headaches), with no evidence of any neurodegenerative disease. The demographic characteristics of each participant group are shown in the Supporting Information Table S1. Twenty-four patients with LBD, three patients with 4R-Tau and nine patients with MSA were treated with antiparkinsonian drugs (levodopa known as L-DOPA, peripheral decarboxylase inhibitors). Therefore, the daily dose of L-DOPA in milligrams per day was taken into account. The group of treated patients included those on either L-DOPA monotherapy or a combination of L-DOPA and a dopamine agonist. In combination pharmacotherapy, only the daily dose of L-DOPA was also taken into account since no effect on the concentration of TRP metabolites was expected for the dopamine agonist. Members of the AD and HC groups were not medicated. The study was not blinded.

### 5.3 Sample Preparation

Blood and CSF collection was performed at 10:00 a.m. with a prior 18 h fasting period. Approximately 10 mL of peripheral blood and CSF were collected by venipuncture or lumbal puncture into sterile tubes (no anticoagulant) under standardized conditions.^73,74^ All samples were processed within 10 min of collection. Blood and CSF were centrifuged at 1100*g* for 10 min at 4 °C. The serum was transferred into dark amber glass vials, heated in a water bath (30 °C for 5 min), sonicated (5 min), and bubbled with a stream of argon (2 min). CSF and serum samples were then immediately stored in the dark at −80 °C until preparation for analysis (long-term stability of the analytes was tested in reference^2^). There was only one freeze–thaw cycle before the analysis.

Levels of neuroactive compounds were determined using a previously published, fully validated, highly sensitive and efficient method.^2^ Briefly, cooled CSF or serum samples (100 μL) were spiked with a predefined quantity of stable isotopically labeled internal standards. Samples were placed in a CoolBox (Biocision) during all pipetting steps and protected from light during processing. Complete precipitation of proteins was induced by incubating (60 min, −20 °C) the samples on a rotator with ice-cold methanol (−20 °C). The samples were then centrifuged at 6.500 rpm for 7 min at 4 °C. Before further centrifugation (8.000 rpm for 5 min at 4 °C), the supernatant was transferred to a microspin centrifuge filter tube with a nylon membrane (pore size, 0.20 μm). The resulting filtrate was evaporated under a stream of nitrogen to dryness. Before analysis, the sample was dissolved in 30 μL of 2% methanol, mixed (30 s), sonicated (5 min), and transferred to a vial insert. Target analytes were quantitated using matrix-matched calibration curves prepared using artificial serum (4% bovine serum albumin in 10 mM phosphate-buffered saline, pH 7.4) or a CSF calibrator.

### 5.4 LC–MS/MS Conditions

The prepared samples were analyzed by UHPLC–MS/MS using an Acquity UPLC (Waters) system connected to a triple quadrupole mass spectrometer Xevo TQ (Waters) with positive electrospray ionization. Samples were stored in an autosampler maintained at 8 °C during analysis and were injected (10 μL) into a reversed-phase chromatography column (Acquity UPLC HSS T3 Column, 100 Å, 1.8 μm, 2.1 mm × 100 mm; Waters) equipped with the appropriate precolumn (VanGuard HSS 1.8 μm; Waters). Mobile phase A was 0.1% formic acid in water, while mobile phase B was methanol. The column was maintained at 30 °C and samples were eluted at a flow rate of 0.3 mL/min using the following gradient: 0–2 min, 98:2 (A:B; isocratic elution); 2–10 min, 40:60 (A:B; gradient elution). A wash step and equilibration were performed at the end of the gradient. The total analytical run time was 14 min. The mass spectrometer was operated in multireaction monitoring mode using the previously reported parameters.^2^ Quantitative analysis was performed using the MassLynx 4.2 (Waters) and Microsoft Office (Microsoft) software packages.

### 4.5 Data Treatment and Statistical Analysis

Statistical analyses were performed in GraphPad (version 9.5, San Diego, California, USA), R (version 4.2.0), and TIBCO Statistica (version 14.0.0, Palo Alto, California, USA). Zero imputation was done using the k-nearest neighbors algorithm as implemented in the R impute package (Hastie, T.; Tibshirani, R.; Narasimhan, B.; Chu, G. impute: Imputation for microarray data, R package version 1.72.3, 2023) with *k* (the number of neighbors used for imputation) being set to 5. The raw data were log-transformed to obtain a normal distribution, and the Shapiro–Wilk test was used to assess normality. Subsequent analyses were based on box-plots, ANOVA with post hoc testing (Holm–Shidak multiple comparisons test), Pearson correlations, and the Mann–Whitney *U* test (for sample groups where normality was not achieved). The *p*-value threshold for significance was <0.05. The study design was planned for a minimum of 10 samples per experimental group to ensure sufficient statistical significance. The power of the study was evaluated, and an effect size of >0.89 (Cohen’s D) for comparisons between studied groups (*n* = 10–31) was found to be statistically significant for a two-tailed *t* test based on type I error (Alpha = 0.05) and the required power (1 – beta = 0.8). Statistically significant correlations in the range 0.35–0.58 were computed for all studied groups (*n* = 10–31).

## Data Availability

The data that support the findings of this study are available from the corresponding author upon reasonable request.

## Supporting Information Available

Figure S1 showing scheme of l-tryptophan metabolism; Table S1 listing the characteristics of the study participants (PDF)

## Abbreviations

3-OH-AA: 3-hydroxyanthranilic acid
3-OH-KYN: 3-hydroxy-l-kynurenine
4R-Tau: four-repeat tauopathy
5-MeO-TA: 5-methoxytryptamine
5-OH-IAA: 5-hydroxyindole-3-acetic acid
5-OH-TRP: 5-hydroxy-l-tryptophan
6-OH-M: 6-hydroxymelatonin
AADC: aromatic amino acid decarboxylase
AA: anthranilic acid
AD: Alzheimer’s disease
AFMK: *N*^1^-acetyl-*N*^2^-formyl-5-methoxykynuramine
*bv*FTD: behavioral variant frontotemporal dementia
CBS: corticobasal syndrome
CSF: cerebrospinal fluid
DLB: dementia with Lewy bodies
DWI-MRI: diffusion-weighted MRI
FLAIR: fluid-attenuated inversion recovery
HC: healthy control
HIS: Hachinski ischemic score
IAA: indole-3-acetic acid
L-DOPA: levodopa
KA: kynurenic acid
KYN: l-kynurenine
LBD: Lewy body disease
M: melatonin
MRI: magnetic resonance imaging
MSA: multiple system atrophy
*N*-Ac-S: *N*-acetylserotonin
*N*-Me-S: *N*-methylserotonin
*N*-Me-TA: *N*-methyltryptamine
PD: Parkinson’s disease
PET: positron emission tomography
PSP: progressive supranuclear palsy
S: serotonin
TA: tryptamine
TCD: transcranial Doppler
TRP: l-tryptophan
UHPLC–MS/MS: ultrahigh performance liquid chromatography–tandem mass spectrometry
USG: ultrasonography
