# Supplementary material for: Patients with Neurodegenerative Proteinopathies Exhibit Altered Tryptophan Metabolism in the Serum and Cerebrospinal Fluid
Source: ACS Chem Neurosci. 2024 Jan 9;15(3):582–92. doi: 10.1021/acschemneuro.3c00611 (PMC10853934; doi:10.1021/acschemneuro.3c00611)
Supplement: Supplementary file 1 — cn3c00611_si_001.pdf [file cn3c00611_si_001.pdf]

ACS Chemical Neuroscience

Supporting Information

Patients with Neurodegenerative Proteinopathies Exhibit Altered Tryptophan Metabolism in the Serum and Cerebrospinal Fluid

Michal Kaleta[a,b,c*], Eva Hényková[a,b,c], Kateřina Menšíková[b,c], David Friedecký[d], Aleš Kvasnička[d], Kateřina Klíčová[b,c], Dorota Koníčková[b,c], Miroslav Strnad[a,b,c], Petr Kaňovský[b,c], Ondřej Novák[a*]

[a]Laboratory of Growth Regulators, Institute of Experimental Botany of the Czech Academy of Sciences & Palacky University, Šlechtitelů 27, 783 71, Olomouc, Czech Republic

[b]Department of Neurology, University Hospital Olomouc, 779 00 Olomouc, Czech Republic

[c]Department of Neurology, Faculty of Medicine and Dentistry, Palacky University, 779 00 Olomouc, Czech Republic

[d]Laboratory for Inherited Metabolic Disorders, Department of Clinical Biochemistry, University Hospital Olomouc and Faculty of Medicine and Dentistry, Palacky University Olomouc, Zdravotníků 248/7, 779 00 Olomouc, Czech Republic

Correspondence*

Michal Kaleta
E-mail: michal.kaleta@upol.cz

Ondřej Novák
E-mail: novako@ueb.cas.cz

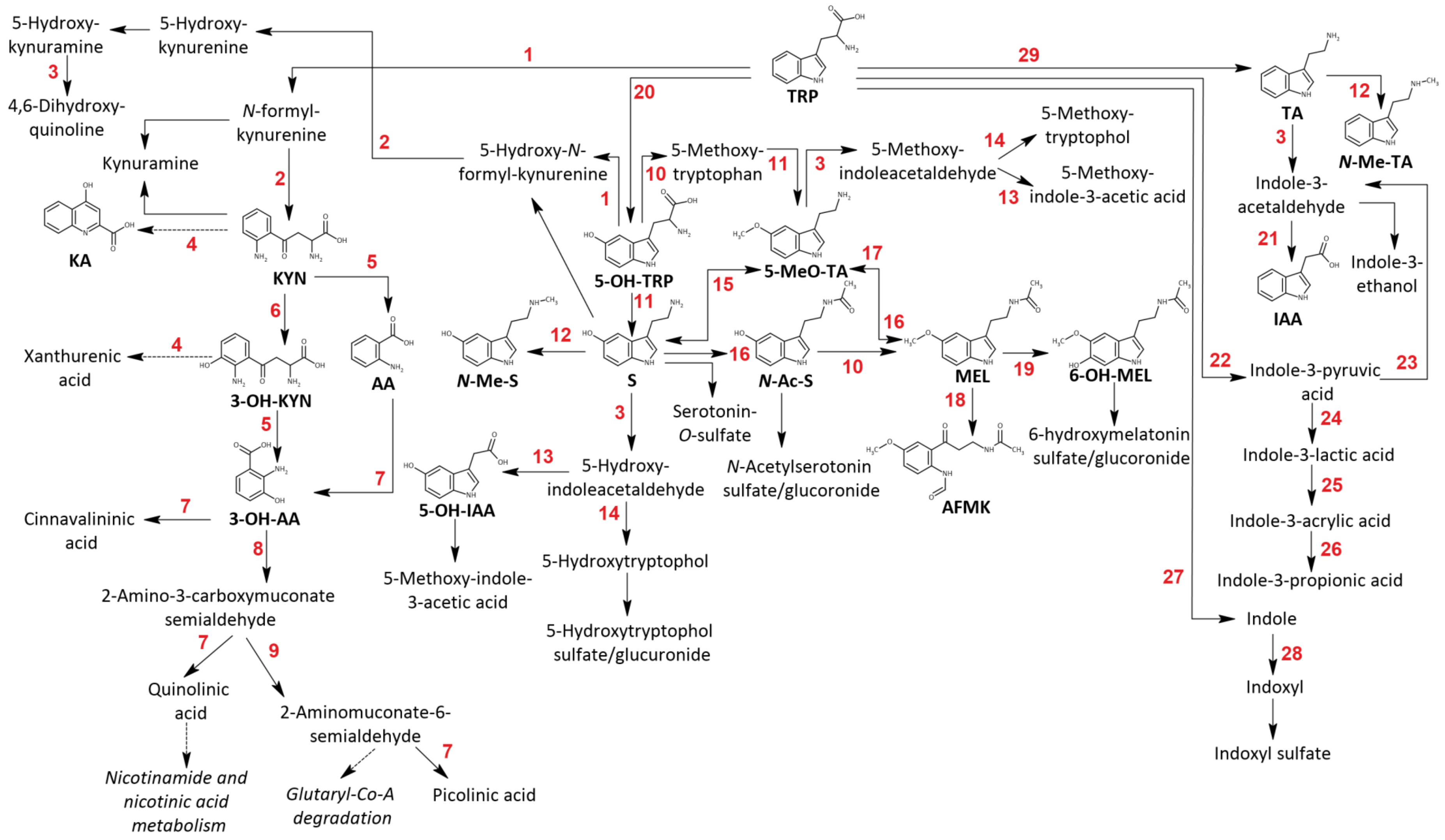

Figure S1. Scheme of L-tryptophan metabolism [1-9]. Metabolites included in the presented study are in bold: 3-OH-AA, 3-Hydroxy-anthranilic acid; 3-OH-KYN, 3-Hydroxy-L-kynurenine; 5-MeO-TA, 5-Methoxytryptamine; 5-OH-IAA, 5-Hydroxyindole-3-acetic acid; 5-OH-TRP, 5-Hydroxy-L-tryptophan; 6-OH-M, 6-Hydroxymelatonin; AA, Anthranilic acid; AFMK, N^1-Acetyl-N^2-formyl-5-methoxykynuramine; IAA, Indole-

3-acetic acid; KA, Kynurenic acid; KYN, L-Kynurenine; M, Melatonin; *N*-Ac-S, *N*-Acetylserotonin; *N*-Me-S, *N*-Methylserotonin; *N*-Me-TA, *N*-Methyltryptamine; S, Serotonin; TA, Tryptamine; TRP, L-Tryptophan. Numbers represent selected enzymes: (1) Indoleamine-2,3-dioxygenase, Tryptophan 2,3-dioxygenase; (2) Formidase; (3) Monoamine oxidase; (4) Kynurenine aminotransferase I-IV; (5) Kynureninase; (6) Kynurenine 3-monooxygenase; (7) Non-enzymatically; (8) 3-Hydroxyanthranillate-3,4-dioxygenase; (9) 2-Amino-3-carboxymuconate-6-semialdehyde decarboxylase; (10) Hydroxyindole-*O*-methyltrasferase; (11) Aromatic amino acid decarboxylase; (12) Indolethylamine *N*-methyltransferase; (13) Aldehyde dehydrogenase; (14) Alcohol dehydrogenase; (15) Methyltransferase; (16) Arylalkylamine *N*-acetyltransferase; (17) Melatonin deacetylase, other aryl acylamidases; (18) Numerous enzymatic, non-enzymatic, free radical, and photochemical mechanisms; (19) CYP1A2, CYP1A1, CYP1B1; (20) Tryptophan hydroxylase; (21) Indole-3-acetaldehyde dehydrogenase; (22) Aromatic amino acid aminotransferase; (23) Indole-3-pyruvic acid decarboxylase; (24) Phenyllactate dehydrogenase; (25) Phenyllactate dehydratase; (26) Acyl-CoA dehydrogenase; (27) Tryptophanase; (28) CYP2E1; (29) Tryptophan decarboxylase

Table S1. Characteristics of the study participants in each group (*n* = 100).

Participants group	Description	Number of participants	Sex ratio (male/female)	Age Median (range)	Mean age at disease onset (years)	Mean duration of disease (years)	L-DOPA therapy (Nr.)	Mean daily L-DOPA dose (mg)
LBD	Parkinson's disease, Dementia with Lewy bodies	31	9/22	69 (38–82)	63.1	4.16	24	577.08
4R-Tau	Progressive supranuclear palsy, Corticobasal syndrome	10	2/8	66 (51–83)	62.6	4.10	3	766.66
MSA	Multiple system atrophy	13	2/11	65 (52–80)	62.2	4.15	9	622.22
AD	Alzheimer's disease	25	3/22	75 (51–90)	70.6	2.64	0	0
HC	Healthy control	21	11/10	57 (37–75)	N/A	N/A	0	0

LBD, Lewy body disease; 4R-Tau, Four-repeat tauopathy; MSA, Multiple system atrophy; AD, Alzheimer's disease; HC, Healthy control; L-DOPA, Levodopa (L-3,4-dihydroxyphenylalanine)

References

(1) Brydges, C.R.; Fiehn, O.; Mayberg, H.S.; Schreiber, H.; Dehkordi, S.M.; Bhattacharyya, S.; Cha, J.; Choi, K.S.; Craighead, W.E.; Krishnan, R.R.; Rush, A.J.; Dunlop, B.W.; Kaddurah-Daouk, R.; Mood Disorders Precision Medicine Consortium. Indoxyl Sulfate, a Gut Microbiome-derived Uremic Toxin, is Associated with Psychic Anxiety and its Functional Magnetic Resonance Imaging-based Neurologic Signature. *Scientific Reports* **2021**, *11* (1), 21011. DOI: 10.1038/s41598-021-99845-1.

(2) Fazio, F.; Lionetto, L.; Curto, M.; Iacovelli, L.; Copeland, C.S.; Neale, S.A.; Bruno, V.; Battaglia, G.; Salt, T.E.; Nicoletti, F. Cinnabarinic Acid and Xanthurenic Acid: Two Kynurenine Metabolites that Interact with Metabotropic Glutamate Receptors. *Neuropharmacology* **2017**, *112*, 365-372. DOI: 10.1016/j.neuropharm.2016.06.020.

(3) Hajsl, M.; Hlavackova, A.; Broulikova, K.; Sramek, M.; Maly, M.; Dyr, J.E.; Suttnar, J. Tryptophan Metabolism, Inflammation, and Oxidative Stress in Patients with Neurovascular Disease. *Metabolites* **2020**, *10* (5), 208. DOI: 10.3390/metabo10050208.

(4) Hardeland, R. Melatonin Metabolism in the Central Nervous System. *Current Neuropharmacology* **2010**, *8* (3), 168-81. DOI: 10.2174/157015910792246244.

(5) Hényková, E.; Vránová, H. P.; Amakorová, P.; Pospíšil, T.; Žukauskaitė, A.; Vlčková, M.; Urbánek, L.; Novák, O.; Mareš, J.; Kaňovský, P.; Strnad, M. Stable Isotope Dilution Ultra-high Performance Liquid Chromatography-tandem Mass Spectrometry Quantitative Profiling of Tryptophan-related Neuroactive Substances in Human Serum and Cerebrospinal Fluid. *Journal of Chromatography A* **2016**, *1437*, 145-157. DOI: 10.1016/j.chroma.2016.02.009.

(6) Liang, Y.; Xie, S.; He, Y.; Xu, M.; Qiao, X.; Zhu, Y.; Wu, W. Kynurenine Pathway Metabolites as Biomarkers in Alzheimer's Disease. *Disease Markers* **2022**, *2022*, 9484217. DOI: 10.1155/2022/9484217.

(7) Roager, H.M.; Licht, T.R. Microbial Tryptophan Catabolites in Health and Disease. *Nature Communications* **2018**, *9*, 3294. DOI: https://doi.org/10.1038/s41467-018-05470-4.

(8) Török, N.; Tanaka, M.; Vécsei, L. Searching for Peripheral Biomarkers in Neurodegenerative Diseases: The Tryptophan-Kynurenine Metabolic Pathway. *International Journal of Molecular Sciences* **2020**, *21* (24), 9338. DOI: 10.3390/ijms21249338.

(9) Wishart, D.S.; Guo, A.; Oler, E.; Wang, F.; Anjum, A.; Peters, H.; Dizon, R.; Sayeeda, Z.; Tian, S.; Lee, B.L.; Berjanskii, M.; Mah, R.; Yamamoto, M.; Jovel, J.; Torres-Calzada, C.; Hiebert-Giesbrecht, M.; Lui, V.W.; Varshavi, D.; Varshavi, D.; Allen, D.; Arndt, D.; Khetarpal, N.; Sivakumaran, A.; Harford, K.; Sanford, S.; Yee, K.; Cao, X.; Budinski, Z.; Liigand, J.; Zhang, L.; Zheng, J.; Mandal, R.; Karu, N.; Dambrova, M.; Schiöth, H.B.; Greiner, R.; Gautam, V. HMDB 5.0: The Human Metabolome Database for 2022. *Nucleic Acids Research* **2022**, *50* (D1), D622-D631. DOI: 10.1093/nar/gkab1062.
